# Systematic Investigation of the Physical and Electrochemical Characteristics of the Vanadium (III) Acidic Electrolyte With Different Concentrations and Related Diffusion Kinetics

**DOI:** 10.3389/fchem.2020.00502

**Published:** 2020-07-14

**Authors:** Minghua Jing, Chengjie Li, Xinyu An, Zeyu Xu, Jianguo Liu, Chuanwei Yan, Dawei Fang, Xinzhuang Fan

**Affiliations:** ^1^Liaoning Engineering Research Center for Advanced Battery Materials, Institute of Metal Research, Chinese Academy of Sciences, Shenyang, China; ^2^Shandong Engineering Research Center of Green and High-Value Marine Fine Chemical, Weifang University of Science and Technology, Shouguang, China; ^3^Institute of Rare and Scattered Elements, College of Chemistry, Liaoning University, Shenyang, China; ^4^Building Energy Research Center, Guangzhou HKUST Fok Ying Tung Research Institute, Guangzhou, China

**Keywords:** temperature, concentration, diffusion equation, trivalent vanadium ion, vanadium flow battery (VFB)

## Abstract

Owing to the lack of systematic kinetic theory about the redox reaction of V(III)/V(II), the poor electrochemical performance of the negative process in vanadium flow batteries limits the overall battery performance to a great extent. As the key factors that influence electrode/electrolyte interfacial reactivity, the physicochemical properties of the V(III) acidic electrolyte play an important role in the redox reaction of V(III)/V(II), hence a systematic investigation of the physical and electrochemical characteristics of V(III) acidic electrolytes with different concentrations and related diffusion kinetics was conducted in this work. It was found that the surface tension and viscosity of the electrolyte increase with increasing V(III) concentration, while the corresponding conductivity shows an opposite trend. Both the surface tension and viscosity change slightly with increasing concentration of H_2_SO_4_, but the conductivity increases significantly, indicating that a lower V(III) concentration and a higher H_2_SO_4_ concentration are conducive to the ion transfer process. The electrochemical measurements further show that a higher V(III) concentration will facilitate the redox reaction of V(III)/V(II), while the increase in H_2_SO_4_ concentration only improves the ion transmission and has little effect on the electron transfer process. Furthermore, the diffusion kinetics of V(III) have been further studied with cyclic voltammetry and chronopotentiometry. The results show that an elevated temperature facilitates the V(III)/V(II) redox reaction and gives rise to an increased electrode reaction rate constant (*k*_s_) and diffusion coefficient [*D*_V(III)_]. On this basis, the diffusion activation energy (13.7 kJ·mol^−1^) and the diffusion equation of V(III) are provided to integrate kinetic theory in the redox reaction of V(III)/V(II).

## Introduction

Vanadium flow batteries (VFBs) have been widely developed as a green energy storage technology because of their high energy efficiency, flexible design, long life cycle, high safety, and low cost (Rychcik and Skyllas-Kazacos, 1988; Sun and Skyllas-Kazacos, 1992; Joerissen et al., 2004; Sukkar and Skyllas-Kazacos, 2004; Zhao et al., 2006; Rahman and Skyllas-Kazacos, 2009; Ding et al., 2013; Chakrabarti et al., 2014; Zheng et al., 2016). In general, VFBs are mainly composed of the electrolyte, electrode, ion exchange membrane, and a bipolar plate. They store energy through the chemical changes in electroactive species, which are separated by the ion exchange membrane (Wang et al., 2014; Xia et al., 2019; Ye et al., 2019, 2020a,b; Yu et al., 2019; Lou et al., 2020). The V(V)/V(IV) and V(III)/V(II) redox couples are used as the catholyte and the anolyte, respectively, and the sulfuric acid solution acts as the supporting electrolyte. The concentrations of vanadium and H^+^ ions play an important role in the determination of the electrochemical reaction processes and the battery performance.

The most commonly used electrolyte in VFBs is an equivalent volume mixture of V(III) and V(IV) sulfuric acid solution. Previous studies have noted that the concentration of V(IV) and acid as well as the operating temperature have important effects on the physicochemical properties and electrochemical activity of the positive electrode reaction (Sum et al., 1985; Kazacos et al., 1990; Zhong and Skyllas-Kazacos, 1992; Iwasa et al., 2003; Yi et al., 2003; Liu et al., 2011). However, few studies have reported on the negative process. Sun and Skyllas-Kazacos (Sum and Skyllas-Kazacos, 1982) investigated the electrochemical behavior of the V(III)/V(II) redox couple at glassy carbon electrodes using cyclic voltammetry (CV). They found that the oxidation/reduction reaction is electrochemically irreversible and the surface preparation is very critical in determining the electrochemical behavior. Yamamura et al. (2005) determined the standard rate constants of the electrode reactions of vanadium on different carbon electrodes. Most studies (Sum and Skyllas-Kazacos, 1982; Oriji et al., 2005; Lee et al., 2012; Aaron et al., 2013; Sun et al., 2016) found that the electrode reaction rate of V(III)/V(II) is much less than that of V(IV)/V(V); however, systematic investigations into the detailed mechanism remain scarce.

Owing to the sluggish kinetics of V(III)/V(II) and the significant hydrogen evolution reaction, the negative process contributes almost 80% polarization during the discharging process (Sun et al., 2016). Agar et al. (2013) further verified that the negative electrode process was the limiting factor in VFB performance by using an asymmetric cell configuration. As the key factors influencing electrode/electrolyte interfacial reactivity, the concentration and physicochemical properties of the V(III) acidic electrolyte as well as the temperature play an important role in the redox reaction of V(III)/V(II) (Xiao et al., 2016). For instance, the viscosity of the electrolyte affects the mass transfer kinetics and the conductivity directly influences the reversibility of the electrochemical reaction, which both depend on the concentration of vanadium and H_2_SO_4_ (Zhang, 2014). In short, it is necessary to conduct a systematic investigation into the physical and electrochemical characteristics of V(III) acidic electrolytes using different concentrations and diffusion kinetics.

In our previous work (Wang et al., 2014), the temperature-related reaction kinetics of the V(IV)/V(V) redox couple on a graphite electrode in sulfuric acid solutions was investigated. Herein, we will investigate the physicochemical properties of the electrolytes with different concentrations of V(III) and sulfuric acid and conduct a systematic study of the diffusion kinetics of V(III). Our aim is to clarify the kinetic rules of the diffusion behavior of V(III) and further establish a diffusion equation, providing a better understanding of the V(III)/V(II) redox reaction in the negative half-cell of VFBs.

## Experimental

### Preparations of the Electrode and Electrolyte

A spectroscopically pure graphite rod (SPGR) (Sinosteel Shanghai Advanced Graphite Material Co. Ltd, China) was used as the working electrode. The working area of the SPGR was ~0.28 cm^2^. This was ground with silicon carbide papers (down to 2,000 grit in grain size) and thoroughly rinsed with deionized water and alcohol before use.

All chemicals used in this work were analytically pure agents and all solutions were prepared with deionized water. V(III) acidic solutions were initially prepared by the electrochemical reduction of VOSO_4_ with an electrolytic cell and then diluted to produce solutions with the required H^+^ and V(III) concentrations. In addition, the concentration of the electrolytes was measured with a ultraviolet spectrometer (TU-1900; Persee General Instrument Co. Ltd, Beijing, China).

### Physical Characterization of the Electrolyte

The viscosity was measured by means of an Ubbelohde viscometer. The electrical conductivity was determined using a conductivity meter (Mettler Toledo) at 293 K. The surface tensions of the solutions were measured by the bubble-pressure method.

### Electrochemical Measurements

The electrochemical measurements were performed using a Reference 600 electrochemical workstation (Gamry Instruments, USA) with a conventional three-electrode cell with an SPGR as the working electrode, a platinum plate as the counter electrode, and a saturated calomel electrode as the reference electrode. A salt bridge was used to eliminate the liquid junction potential between the Luggin capillary and the working electrode. The electrolyte was purged with nitrogen for 10 min before the electrochemical test to reduce the influence of oxygen on the electrochemical oxidation of V(II). Temperature was controlled by a water bath.

## Results and Discussion

### Physical Characteristics of the V(III) Acidic Electrolyte at Different Concentrations

The physical parameters of the electrolyte, such as the surface tension, viscosity, and conductivity, significantly affect the ion transmission process and the electrochemical properties of the electrode/electrolyte interface (Jing et al., 2016). In general, higher surface tension will hinder the contact between the electrolyte and the electrode, leading to a decrease in the effective reaction area, while higher conductivity often means faster transmission of ions and higher viscosity usually leads to a lower diffusion rate. However, the three physical parameters are not all proportional to the concentration of the electrolyte, so it is not reasonable to simply increase or decrease the electrolyte concentration in engineering applications. In particular, vanadium ions often exist in a very complex form in the electrolyte, which may result in a significant difference in the physicochemical properties of the electrolyte at different concentrations (Sepehr and Paddison, 2016). Hence, it is necessary to investigate the influence of the electrolyte concentration on its physicochemical properties.

**Figure 1:**
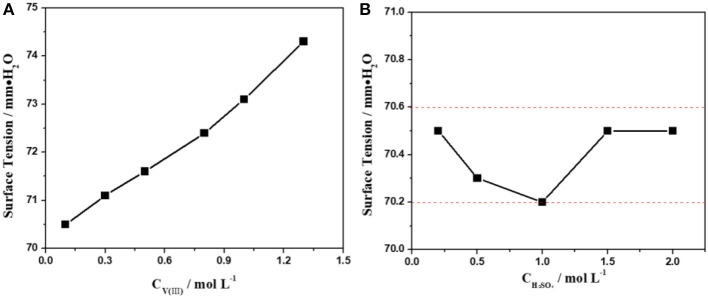
Changes in the surface tension with different concentrations of V(III) **(A)** and H_2_SO_4_ **(B)**.

The influence of the concentration of V(III) and H_2_SO_4_ on the surface tension of the electrolyte was investigated first. Figure 1A shows the surface tension of 2.0 mol·L^−1^ H_2_SO_4_ solutions with different concentrations of V(III) (from 0.1 to 1.3 mol·L^−1^). Obviously, the surface tension of the electrolytes gradually increases with an increasing concentration of V(III). Actually, the higher the concentration of vanadium, the higher the surface tension and the greater the effect on the contact between the electrolyte and the electrode. However, in practice, we want to increase the concentration of vanadium to achieve high volumetric capacities or energy densities. We can resolve this contradiction by improving the hydrophilicity of the electrode surface to apply a higher concentration of vanadium. In contrast to Figure 1A, the variation in the trend in the surface tension was very slight when changing the H_2_SO_4_ concentration (Figure 1B). Such a different phenomenon might be attributed to the stronger hydration force of the V(III) compared with that of H_2_SO_4_. Therefore, the surface tension of the V(III) acidic electrolyte was mainly affected by the concentration of V(III).

**Figure 2:**
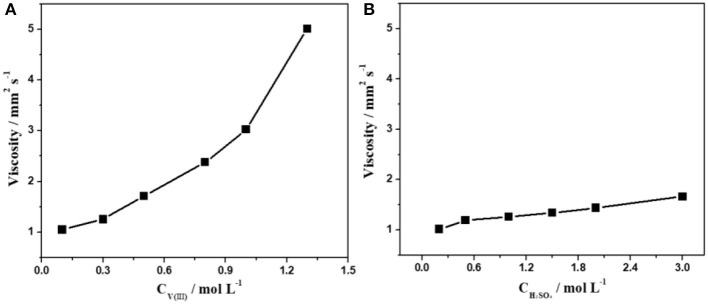
Changes in the viscosity with different concentrations of V(III) **(A)** and H_2_SO_4_ **(B)**.

The influence of the concentration of V(III) and H_2_SO_4_ on the viscosity and conductivity of the electrolyte was also investigated. As shown in Figure 2, there was a fourfold increase in the viscosity as the concentration of V(III) changed from 0.1 to 1.3 mol·L^−1^. However, the viscosity of the electrolytes changed very little with different concentrations of H_2_SO_4_, which was similar to the changing features of the surface tension described above. This could be ascribed to the more complex structure of V(III). It can be concluded that the concentration of V(III) was the main factor in determining the viscosity of the V(III) acid electrolytes, and a suitable concentration of V(III) had a positive effect on its mass transfer performance.

**Figure 3:**
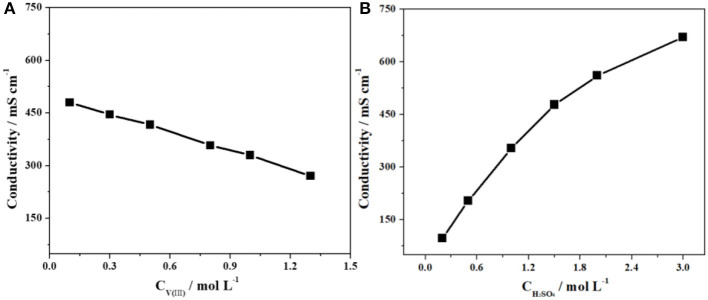
Changes in conductivity with different concentrations of V(III) **(A)** and H_2_SO_4_ **(B)**.

Figure 3 shows the variations in the conductivity of the electrolytes with different concentrations of V(III) (a) and H_2_SO_4_ (b). When the concentration of V(III) increased from 0.1 to 1.3 mol·L^−1^, the conductivity decreased by ~43.3% (from 480 to 272 mS·cm^−1^), while there was an obvious increase in conductivity (about 6-fold) with increasing H_2_SO_4_ concentration. The significant difference should also be ascribed to the more complex form of V(III), which would result in a larger hydrated ionic radius and poorer mobility (Sepehr and Paddison, 2016). Therefore, it is necessary to investigate the optimum concentration of V(III) and H_2_SO_4_ to obtain better electrochemical performance.

### Electrochemical Characteristics of the V(III) Acidic Electrolyte at Different Concentrations

The CV test was a useful tool to investigate the electrochemical performance of the battery materials. For a CV curve, the value of the peak currents of the oxidation and reduction reactions (*i*_*pa*_, *i*_*pc*_, respectively) and their ratio (–*i*_*pa*_*/i*_*pc*_), as well as the peak potential separation (Δ*E*_*p*_), could be used to estimate the electrochemical activity. Generally, the lower the value of Δ*E*_*p*_ or the more similar the *i*_*pa*_ and –*i*_*pc*_ values usually implied better electrochemical reversibility, and a higher peak current often suggested higher reactivity (Bard and Faulkner, 2001; Ding et al., 2013). However, the peak current (*i*_*pc*_) is closely related to the electrochemical surface area of the electrode, which is difficult to read directly from the CV curve, so Δ*E*_*p*_ and –*i*_*pa*_/*i*_*pc*_ were more suitable for estimating the electrochemical properties.

Specifically, –*i*_*pa*_*/i*_*pc*_ can be calculated from the CV curves by the following equation (Bard and Faulkner, 2001):

(1) $$\begin{array}{r} \frac{i_{pc}}{i_{pa}}=\frac{{\left(i_{pc}\right)}_{0}}{i_{pa}}+\frac{0.485{\left(i_{sp}\right)}_{0}}{i_{pa}}+0.086 \end{array}$$

where (*i*_*pc*_)_0_ is the uncorrected cathodic peak current density with respect to the zero current baseline and (*i*_*sp*_)_0_ is the current density at the switching potential.

**Figure 4:**
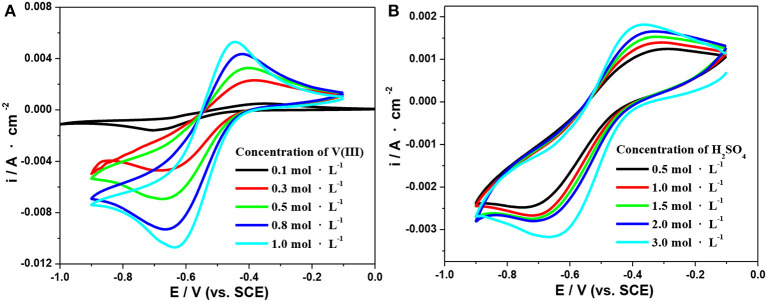
CV curves on an SPGR in 2.0 mol·L^−1^ H_2_SO_4_ with different concentrations of V(III) **(A)** and 0.1 mol·L^−1^ V(III) with different concentrations of H_2_SO_4_ **(B)**. Scan rate, 10 mV·s^−1^.

Herein, CV tests on the SPGR in 2.0 mol·L^−1^ H_2_SO_4_ with different concentrations of V(III) electrolytes (from 0.1 to 1.0 mol·L^−1^) were first carried out at a scan rate of 10 mV·s^−1^ (Figure 4A). The detailed electrochemical parameters are listed in Table 1. As expected, the value of –*i*_*pa*_*/i*_*pc*_ gradually increased to 0.97 with increasing concentration of V(III), indicating a favorable electrochemical reversibility of V(III)/V(II) with a 1.0 mol·L^−1^ V(III) acidic electrolyte. In addition, Δ*E*_*p*_ gradually decreased with increasing concentration of V(III), also indicating increasing electrochemical activity of the electrolyte with higher concentrations of V(III). It can be concluded that a higher concentration of V(III) would facilitate the V(III)/V(II) electrochemical redox reaction.

**Table 1:** Electrochemical parameters recorded from the CV curves in Figure 4.

***C*_**V(III)**_ (mol·L^**−1**^)**	***–i_***pa***_/i_***pc***_***	***ΔE_***p***_*/V**	***C*H_**2**_SO_**4**_ (mol·L^**−1**^)**	***–i_***pa***_/i_***pc***_***	***ΔE_***p***_*/V**
0.1	0.914	0.354	0.5	0.905	0.464
0.3	0.929	0.307	1.0	0.911	0.414
0.5	0.940	0.274	1.5	0.916	0.397
0.8	0.959	0.243	2.0	0.919	0.391
1.0	0.970	0.189	3.0	0.912	0.304

Next, CV tests on the SPGR in 0.1 mol·L^−1^ V(III) with different concentrations of H_2_SO_4_ electrolytes (from 0.5 to 3.0 mol·L^−1^) were carried out at a scan rate of 10 mV·s^−1^. The corresponding CV curves are shown in Figure 4B; the detailed electrochemical parameters recorded from the CV curves in Figure 4B are also listed in Table 1. Compared with the CV curves in Figure 4A, the CV curves in the electrolytes at different H_2_SO_4_ concentrations showed a smaller difference. Even so, the value of Δ*E*_*p*_ obviously decreased with increasing concentration of H_2_SO_4_, which should be ascribed to the rapid transfer rate of H^+^, resulting in favorable conductivity of the electrolytes with higher concentrations of H_2_SO_4_. In addition, the value of –*i*_*pa*_*/i*_*pc*_ increased first and then decreased, and the maximum value was obtained with 2.0 mol·L^−1^ H_2_SO_4_ electrolyte, which might be attributed to the coupling effect of both the increased conductivity and viscosity as well as the gradually increasing influence of the hydrogen evolution reaction with increasing H_2_SO_4_ concentration. However, the –*i*_*pa*_*/i*_*pc*_ values changed little with H_2_SO_4_ concentration, indicating that the H_2_SO_4_ concentration had a smaller effect on the electron transfer process.

Electrochemical impedance spectroscopy (EIS) is a powerful non-destructive technique for studying the electrochemical processes at the electrode/electrolyte interface. Electrochemical parameters such as the solution resistance (*R*_s_), constant resistance (*R*_c_), and electron transfer resistance (*R*_ct_) can be obtained simultaneously through the appropriate equivalent circuit (Cao and Zhang, 2002).

**Figure 5:**
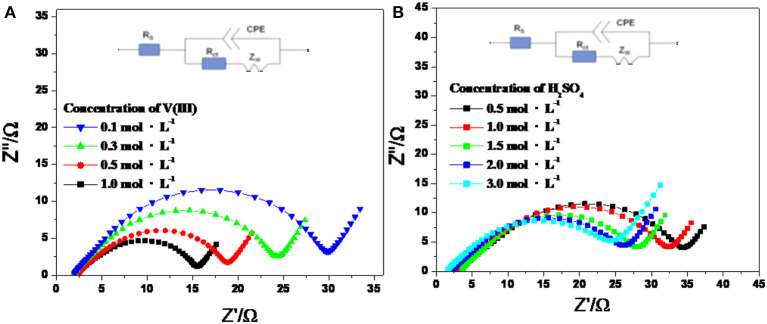
Nyquist plots and the equivalent electric circuit of V(III)/V(II) redox couples with 2.0 mol·L^−1^ H_2_SO_4_ electrolyte with different concentrations of V(III) **(A)** and 0.1 mol·L^−1^ V(III) electrolyte with different concentrations of H_2_SO_4_ **(B)** at a polarization potential of −0.6 V.

Figure 5 shows the Nyquist plots of an SPGR recorded in different electrolytes at a polarization potential of −0.6 V, with an excitation signal of 5 mV and frequency ranging from 0.1 mHz to 10 mHz. As shown in Figure 5, the Nyquist plots for all samples consisted of a semicircle at high frequency and a linear part at low frequency, suggesting that the electrode reaction was dual controlled by the electrochemical reaction and diffusion processes (Wei et al., 2014). Thus, the Nyquist plots in Figure 5 also show the equivalent circuits, where *R*_s_ is the bulk solution resistance; CPE is the constant phase element, which accounts for the double-layer capacitance; *R*_ct_ signifies the faradaic interfacial charge-transfer resistance; and *Z*_w_ is the diffusion capacitance attributed to the diffusion process of vanadium ions (Wang and Wang, 2007; Wei et al., 2014).

According to the fitting results in Table 2, *R*_s_ increased gradually with increasing V(III) concentration, which was caused by the decreased conductivity of the electrolytes. *R*_ct_ decreased dramatically with increasing concentration of V(III), indicating the better electrochemical reactivity of a higher concentration of V(III), which was consistent with the CV results. Comparing the increased *R*_s_ with the decreased *R*_ct_, the latter was much more remarkable, thus the electrochemical polarization was more prominent than the ohmic polarization on the SPGR in the V(III) acid electrolytes. For the electrolytes with different H_2_SO_4_ concentrations, *R*_s_ rapidly decreased with increasing H_2_SO_4_ concentration, owing to the greater conductivity of the electrolyte with a higher concentration of H^+^. However, *R*_ct_ was almost unchanged with increasing H_2_SO_4_ concentration, suggesting that the electron transfer process of the V(III)/V(II) redox reaction had little relationship with H^+^. In short, the concentration of H_2_SO_4_ mainly affected the ohmic resistance of the electrolyte, while the V(III) concentration mostly influenced its electron transfer resistance, which was consistent with the CV results.

**Table 2:** EIS parameters obtained by fitting the impedance plots with the equivalent electric circuits in Figure 5.

***C*_**V(III)**_ (mol·L^**−1**^)**	***R*_**s**_ (Ω)**	***R*_**ct**_ (Ω)**	***C*H_**2**_SO_**4**_ (mol·L^**−1**^)**	***R*_**s**_ (Ω)**	***R*_**ct**_ (Ω)**
0.1	1.891	24.11	0.5	4.594	18.23
0.3	1.988	18.58	1.0	3.34	21.75
0.5	2.283	8.899	1.5	2.51	20.39
1.0	2.389	8.28	2.0	2.072	19.47
–	–	–	3.0	1.053	19.5

**Figure 6:**
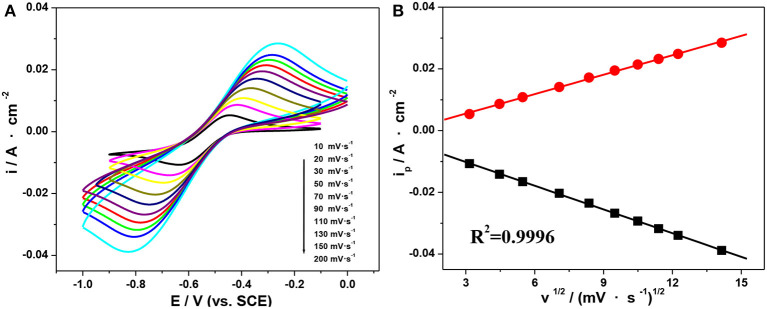
CV curves on an SPGR recorded at different scan rates in 1.0 mol·L^−1^ V(III) with 2.0 mol·L^−1^ H_2_SO_4_ **(A)**. Peak current density as a function of the square root of the scan rate **(B)**.

Based on the above, the electrolyte containing 1.0 mol·L^−1^ V(III) and 2.0 mol·L^−1^ H_2_SO_4_ exhibited favorable electrochemical properties, so CV behaviors at different scan rates in that electrolyte were further investigated. As shown in Figure 6A, the oxidation and reduction peaks showed comparative symmetry at all scan rates, indicating a favorable electrochemical reversibility. In addition, the peak current proved to be proportional to the square root of the scan rate (Figure 6B), which suggested that the oxidation and reduction reaction of the V(III)/V(II) redox couples on an SPGR were controlled by the diffusion process (Wei et al., 2014).

### Diffusion Kinetics Study of the V(III) Acid Electrolytes

CV is one of the most commonly used electrochemical techniques to study the electrode reaction kinetics. For an irreversible electrode process, the peak current density is given by Bard and Faulkner (2001):

(2) $$\begin{array}{r} i_{p}=4.958\times 10^{-4}nFC_{b}D^{1/2}V^{1/2}{\left(-\alpha n_{\alpha }F/RT\right)}^{1/2} \end{array}$$

where *V* is the potential sweep rate (V·s^−1^) and *D* is the diffusion coefficient of the active reactant (cm^2^·s^−1^). Based on Equation (2), we can obtain the value of *D*_V(III)_ at different temperatures from the slope of the plot of *i*_*p*_ vs. *V*^1/2^.

Moreover, the values of the reaction rate constant *k*_s_ can be calculated by Equation (3) (Bard and Faulkner, 2001):

(3) $$\begin{array}{r} i_{p}=2.27\times 10^{-4}nFC_{b}k_{s}\exp\left[-\alpha n_{\alpha }F\left(E_{P}-E^{O^{\prime }}\right)/RT\right] \end{array}$$

where *i*_*p*_ is the peak current density (A·cm^−2^); *E*_*p*_ is the peak potential (V); *C*_*b*_ is the bulk concentration of the electroactive species (mol·L^−1^); *k*_*s*_ is the standard heterogeneous rate constant (cm·s^−1^); α is the charge transfer coefficient; *E*^*O*^′ is the formal potential of the electrode; *n* is the number of electrons involved in the rate-limiting step; and other symbols such as *F, R*, and *T* have their usual meanings.

The formal potential *E*^*O*^′ at different temperatures can be calculated by Equation (4) (Bard and Faulkner, 2001):

(4) $$\begin{array}{r} {E^{∙}}^{\prime }=\frac{\overset{j}{\underset{i=1}{\sum }}\left(E_{pa_{j}}+E_{pcj}\right)/2}{j} \end{array}$$

where *j* is the total number of potential scans applied in the CV tests; *E*_*pa*_ is the anodic peak potential; and *E*_*pc*_ is the cathodic peak potential.

**Figure 7:**
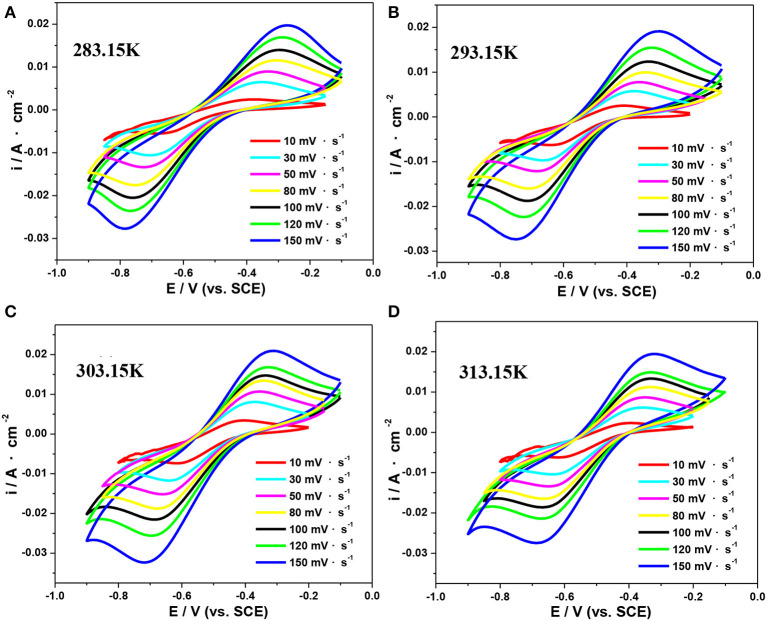
CV curves of an SPGR in 0.5 mol·L^−1^ V(III) with 2.0 mol·L^−1^ H_2_SO_4_ electrolyte at 283.15 K **(A)**, 293.15 K **(B)**, 303.15 K **(C)**, and 313.15 K **(D)**.

Herein, CV tests in an electrolyte consisting of 0.5 mol·L^−1^ V(III) with 2.0 mol·L^−1^ H_2_SO_4_ at different temperatures were conducted to study their electrode reaction kinetics. Figure 7 shows the typical CV curves at scan rates ranging from 10 to 200 mV·s^−1^ at 283.15, 293.15, 303.15, and 313.15 K, respectively. As shown in Figure 7, Δ*E*_*p*_ was significantly >60 mV, indicating the electrochemical irreversibility of the V(III)/V(II) redox reaction (Kazacos et al., 1990; Aaron et al., 2013).

The mean values of –*i*_*pc*_*/i*_*pa*_ and the formal potential *E*^*O*^′ at different scan rates obtained from the CV curves in Figure 7 are listed in Table 3. The values of –*i*_*pc*_*/i*_*pa*_ changed slightly with the temperature, suggesting an insignificant effect of temperature on the reversibility of the V(III)/V(II) redox reaction.

**Table 3:** The mean values of –*i*_*pc*_*/i*_*pa*_ and the formal potential (*E*^*O*^′) calculated from the CV curves in Figure 7.

***T* (K)**	***–i_***pc***_/i_***pa***_***	***E^*O*^^′^* (V)**
283.15	1.48	−0.529
293.15	1.53	−0.524
303.15	1.49	−0.511
313.15	1.41	−0.509

**Figure 8:**
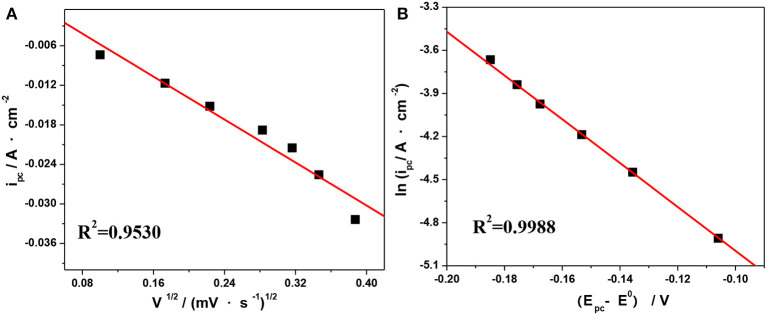
Plots of *i*_*pc*_ vs. *V*^1/2^ **(A)** and ln *i*_*pa*_ vs. (*E*_*pc*_–*E*^*O*^′) **(B)** at 303.15 K from the CV curves in Figure 7C.

The values of the anodic charge transfer coeffcient (α) and electron transfer number (*n*) have been estimated to be 0.56 and 1, respectively, according to our earlier work (Jing, 2017). Based on Equation (2–3) in Jing (2017), we can deduce a linear relationship between *i*_*pc*_ vs. *V*^1/2^ and ln *i*_*pc*_ vs. (*E*_*p*_*- E*^*O*^′). The corresponding results measured at 303.15 K are shown in Figures 8A,B. Next, the values of *D*_V(III)_ and *k*_*s*_ at different temperatures can be calculated according to the slope of the linear curves in Figures 8A,B, respectively. For comparison, the viscosities (η) measured by Ubbelohde viscometry under different temperatures are listed in Table 4.

**Table 4:** Rate constant of the reduction reaction (*k*_*s*_) and the diffusion coefficient (*D*_V(III)_) of V(III) at different temperatures.

***T* (K)**	***D*_V(III)_ (10^−7^ cm^2^·s^−1^)**	***k_***s***_* (10^**−5**^ cm·s^**−1**^)**	**η (mm^2^·s^−1^)**
283.15	5.03	6.84	2.22
293.15	5.53	14.2	1.71
303.15	8.27	16.6	1.37
313.15	11.9	26.6	1.13

The results in Table 4 show that the values of the reaction rate constants (*k*_*s*_) were of the order of 10^−5^ cm·s^−1^ and became larger with increasing temperature, which suggested that a higher temperature might facilitate the V(III)/V(II) redox reaction. Furthermore, *D*_V(III)_ increased from 5.034 × 10^−7^ cm^2^·s^−1^ at 283.15 K to 11.9 × 10^−7^ cm^2^·s^−1^ at 313.15 K, suggesting that an increased temperature was beneficial to the mass transfer of V(III), which was also reflected in the change of viscosity. Indeed, the diffusion coefficient of the active ion has an important effect on the battery performance. The larger coefficient suggests a faster ion migration rate, which is conducive to the mass transfer kinetics of the electrode reaction reducing the concentration polarization of the battery under a higher current density and leading to a better rate capability and electrolyte utilization rate.

**Figure 9:**
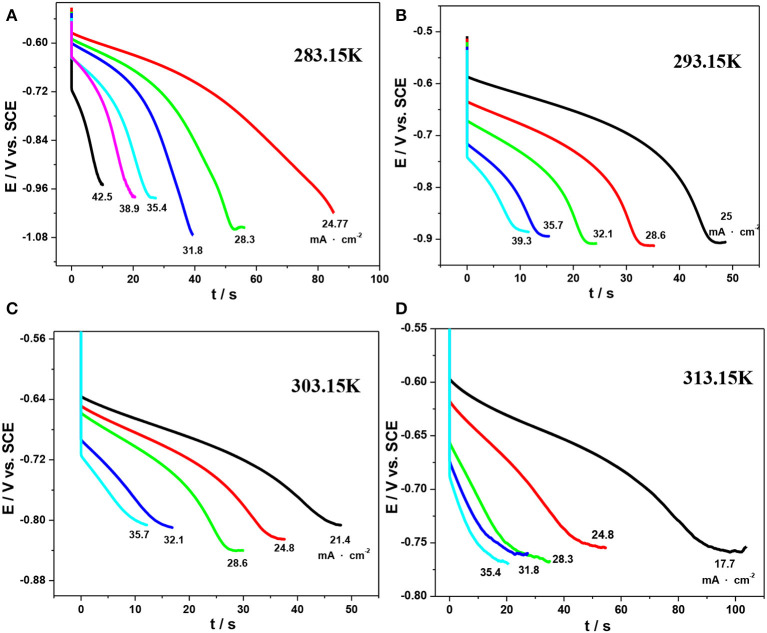
Chronopotentiograms for the reduction reaction of V(III) on an SPGR in 1.0 mol·L^−1^ V(III) with 2.0 mol·L^−1^ H_2_SO_4_ at various temperatures: 283.15 K **(A)**, 293.15 K **(B)**, 303.15 K **(C)**, and 313.15 K **(D)**.

However, as mentioned above, CV is not an ideal quantitative method to determine the kinetic parameters of the peak current. Herein, chronopotentiometry was carried out as it is a promising approach to obtain the diffusion coefficient by Sand's equation (Kazacos et al., 1990; Sepehr and Paddison, 2016). The corresponding potential–time curves under various temperatures are shown in Figure 9.

For an irreversible or reversible reaction, Sand's equation is given by Kazacos et al. (1990):

(5) $$\begin{array}{r} \tau =\frac{n^{2}F^{2}\pi DC_{b}^{2}}{4i^{2}} \end{array}$$

where τ is the total time taken to achieve an abrupt change in the potential of the electrode and *i* is the current density. The values of τ are determined as the transition time when the absolute values of the slope of the plots increase abruptly.

**Figure 10:**
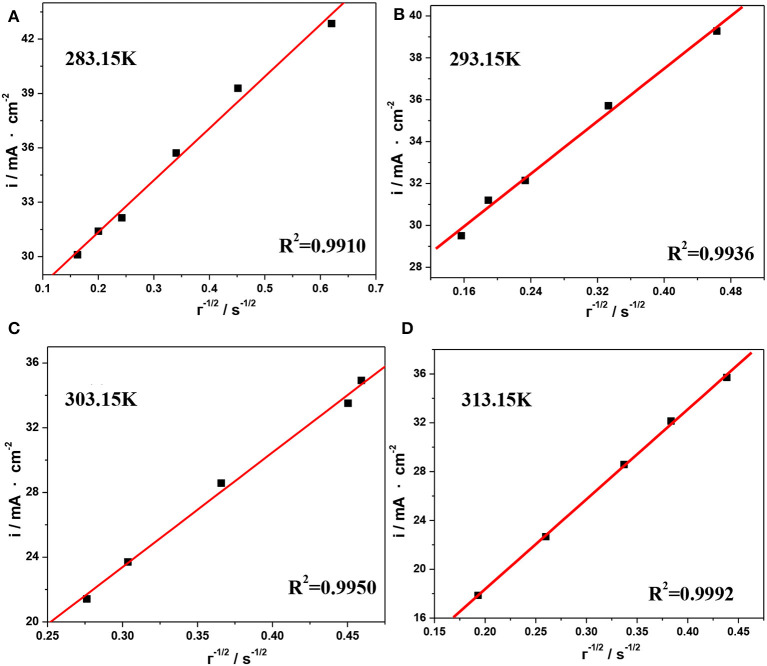
Current density as a function of the reciprocal of the square root of the transition time for the reduction reaction of V(III) at different temperatures, **(A)** 283.15 K, **(B)** 293.15 K, **(C)** 303.15 K, and **(D)** 313.15 K.

The plots of *i* vs. τ^1/2^ obtained from Figure 9 are shown in Figure 10, and the values of *D*_V(III)_ calculated from the slopes of these plots are listed in Table 5. By comparing the *D*_V(III)_ values in Tables 4, 5, it can be seen that the values of *D*_V(III)_ obtained from CV and chronopotentiometry were of the same order (10^−7^ cm^2^·s^−1^), but there was a smaller variability when changing the temperature, which might result in a smaller deviation of the calculated *D*_V(III)_ values.

**Table 5:** Diffusion coefficients of V(III) ions calculated from Figure 10 at different temperatures.

***T* (K)**	***D*_V(III)_ (10^−7^ cm^2^·s^−1^)**
283.15	1.09
293.15	1.30
303.15	1.60
313.15	1.95

Based on the chronopotentiometry results, the diffusion activation energy, *E*_*D*_, can be obtained from the slope of the plot of ln *D(T)* vs. *1/T* by the Arrhenius equation (Zha, 2002):

(6) $$\begin{array}{r} D\left(T\right)=D_{0}\exp\left(-E_{D}/RT\right) \end{array}$$

where *D*_0_ is a temperature-independent factor (cm^2^·s^−1^) and *E*_*D*_ is the diffusion activation energy.

**Figure 11:**
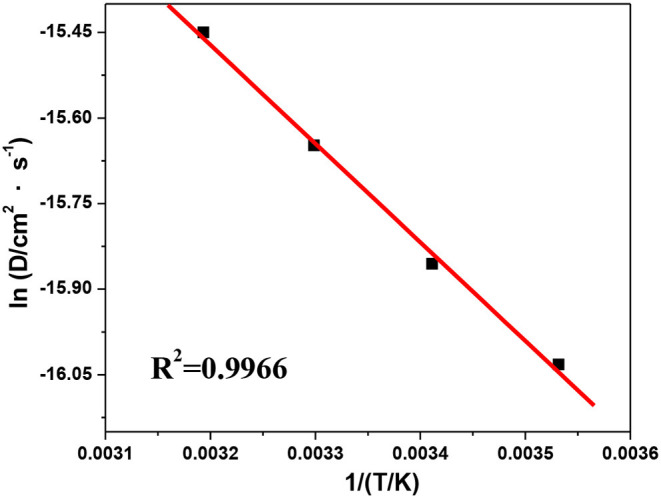
Plot of ln *D(T)* vs. *1/T*.

The shift of ln *D(T)* with *1/T* is shown in Figure 11, from where the values of *E*_*D*_ and *D*_0_ can be estimated as 13.7 kJ·mol^−1^ and 1.3 × 10^−4^ cm^2^·s^−1^, respectively. As a result, the diffusion coefficient of V(III) can be expressed as follows:

(7) $$\begin{array}{r} D_{\text{V}\left(\text{III}\right)}=1.3\times 10^{-4}\exp13700/RT \end{array}$$

which could be used to estimate the diffusion behavior of V(III). In summary, an increase in temperature could facilitate the V(III)/V(II) redox reaction and improve the mobility of V(III) ions in the negative electrolyte, which would result in improved electrochemcial performance. However, the more intense hydrogen evolution at higher temperatures should also be considered.

## Conclusion

In this work, the physical and electrochemical characteristics of the V(III) acidic electrolytes at different concentrations and with different diffusion kinetics have been systemically investigated. The results show that the surface tension and viscosity of the V(III) acidic electrolyte were mainly affected by the V(III) concentration and that they were in direct proportion to each other, which suggested the negative effects of a high concentration of V(III) on the mass transfer kinetics. As the supporting electrolyte, the H_2_SO_4_ concentration had a significant effect on the conductivity of the electrolyte; however, the higher H_2_SO_4_ concentration might result in significant hydrogen evolution and increased mass transfer resistance. The electrochemical measurements showed that a higher V(III) concentration would facilitate the redox reactions of V(III)/V(II), while the increase in H_2_SO_4_ concentration could improve the ion transmission and had little effect on the electron transfer process. In addition, the diffusion kinetics of V(III) were further studied by CV and the chronopotentiometry method. The results demonstrated that an elevated temperature would facilitate the V(III)/V(II) redox reaction, and so the reaction rate constant (*k*_*s*_) and diffusion coefficient [*D*_V(III)_] were obtained at different temperatures. On this basis, the diffusion activation energy (13.7 kJ·mol^−1^) and the diffusion equation for V(III) are provided to integrate kinetic theory in the redox reaction of V(III)/V(II).

## Data Availability Statement

The raw data supporting the conclusions of this article will be made available by the authors, without undue reservation.

## Author Contributions

MJ, ZX, and XA carried out the experiment. MJ and CL wrote the manuscript with support from JL and CY. XF conceived the original idea and supervised the project. All authors contributed to the article and approved the submitted version.

## Conflict of Interest

The authors declare that the research was conducted in the absence of any commercial or financial relationships that could be construed as a potential conflict of interest.

## References

[B1] Aaron D. Sun C. Bright M. Papandrew A. Mench M. Zawodzinski T. (2013). In situ kinetics studies in all-vanadium redox flow batteries. ECS Electrochem. Lett. 2, A29–A31. 10.1149/2.001303eel

[B2] Agar E. Dennison C. Knehr K. Kumbur E. (2013). Identification of performance limiting electrode using asymmetric cell configuration in vanadium redox flow batteries. J. Power Sources 225, 89–94. 10.1016/j.jpowsour.2012.10.016

[B3] Bard A. Faulkner L. (2001). Electrochemical Methods-Fundamentals and Applications, 2nd Edn New York, NY: Wiley-Interscience.

[B4] Cao C. Zhang J. (2002). An Introduction to Electrochemical Impedance Spectroscopy. Beijing: Science Press.

[B5] Chakrabarti M. Brandon N. Hajimolana S. Tariq F. Yufit V. Hashim M. (2014). Application of carbon materials in redox flow batteries. J. Power Sources 253, 150–166. 10.1016/j.jpowsour.2013.12.038

[B6] Ding C. Zhang H. Li X. Liu T. Xing F. (2013). Vanadium flow battery for energy storage: prospects and challenges. J. Phys. Chem. Lett. 4, 1281–1294. 10.1021/jz4001032 26282141

[B7] Iwasa S. Wei Y. Fang B. Arai T. Kumagai M. (2003). Electrochemical behavior of the V(IV)/V(V) couple in sulfuric acid medium. Bat. Bimonthly 33, 339–341. Available online at: http://en.cnki.com.cn/Article_en/CJFDTotal-DACI200306022.htm

[B8] Jing M. (2017). Kinetics of the Negative Electrode Processes of the Vanadium Redox Flow Battery and Preparation of Highly Effective Electrode Materials. University of Chinese Academy of Sciences.

[B9] Jing M. Wei Z. Su W. He H. Fan X. Qin Y. (2016). Improved electrochemical performance for vanadium flow battery by optimizing the concentration of the electrolyte. J. Power Sources 324, 215–223. 10.1016/j.jpowsour.2016.05.099

[B10] Joerissen L. Garche J. Fabjan C. Tomazic G. (2004). Possible use of vanadium redox-flow batteries for energy storage in small grids and stand-alone photovoltaic system. J. Power Sources 127, 98–104. 10.1016/j.jpowsour.2003.09.066

[B11] Kazacos M. Cheng M. Skyllas-Kazacos M. (1990). Vanadium redox cell electrolyte optimization studies. J. Appl. Electrochem. 20, 463–467. 27295523

[B12] Lee J. Hong J. Kjeang E. (2012). Electrochemical characteristics of vanadium redox reactions on porous carbon electrodes for microfluidic fuel cell applications. Electrochim. Acta 83, 430–438. 10.1016/j.electacta.2012.07.104

[B13] Liu H. Xu Q. Yan C. Cao Y. Qiao Y. (2011). The effect of temperature on the electrochemical behavior of the V(IV)/V(V) couple on a graphite electrode. Int. J. Electrochem. Sci. 6, 3483–3496. Available online at: http://electrochemsci.org/papers/vol6/6083483.pdf

[B14] Lou X. Yuan D. Yu Y. Lei Y. Ding M. Sun Q.. (2020). A cost-effective nafion composite membrane as an effective vanadium-ion barrier for vanadium redox flow batteries. Chem. Asian J. 15, 1–8. 10.1002/asia.202000140 32166875

[B15] Oriji G. Katayama Y. Miura T. (2005). Investigations on V(IV)/V(V) and V(II)/V(III) redox reactions by various electrochemical methods. J. Power Sources 139, 321–324. 10.1016/j.jpowsour.2004.03.008

[B16] Rahman F. Skyllas-Kazacos M. (2009). Vanadium redox battery: positive half-cell electrolyte studies. J. Power Sources 189, 1212–1219. 10.1016/j.jpowsour.2008.12.113

[B17] Rychcik M. Skyllas-Kazacos M. (1988). Characteristics of a new all-vanadium redox flow battery. J. Power Sources 22, 59–67. 10.1016/0378-7753(88)80005-3

[B18] Sepehr F. Paddison S. (2016). Effect of sulfuric and triflic acids on the hydration of vanadium cations: an *ab initio* study. J. Phys. Chem. A 119, 5749–5761. 10.1021/acs.jpca.5b01794 25954916

[B19] Sukkar T. Skyllas-Kazacos M. (2004). Membrane stability studies for vanadium redox cell applications. J. Appl. Electrochem. 34, 137–145. 10.1023/B:JACH.0000009931.83368.dc

[B20] Sum E. Rychcik M. Skyllas-Kazacos M. (1985). Investigation of the V(V)/V(IV) system for use in the positive half-cell of a redox battery. J. Power Sources 16, 85–95. 10.1016/0378-7753(85)80082-3

[B21] Sum E. Skyllas-Kazacos M. (1982). A study of the V(II)/V(III) redox couple for redox flow cell applications. J. Power Sources 15, 179–190. 10.1016/0378-7753(85)80071-9

[B22] Sun B. Skyllas-Kazacos M. (1992). Modification of graphite electrode materials for vanadium redox flow battery application-I thermal treatment. Electrochim. Acta 37, 1253–1260. 10.1016/0013-4686(92)85064-R

[B23] Sun C. Delnick F. Aaron D. Papandrew A. Mench M. Zawodzinski T. (2016). Probing electrode losses in all-vanadium redox flow batteries with impedance spectroscopy. ECS Electrochem. Lett. 2, A43–A45. 10.1149/2.001305eel

[B24] Wang W. Fan X. Liu J. Yan C. Zeng C. (2014). Temperature-related reaction kinetics of the vanadium (IV)/(V) redox couple in acidic solutions. RSC Adv. 4, 32405–32411. 10.1039/C4RA04278F

[B25] Wang W. Wang X. (2007). Investigation of Ir-modified carbon felt as the positive electrode of an all-vanadium redox flow battery. Electrochim. Acta 52, 6755–6762. 10.1016/j.electacta.2007.04.121

[B26] Wei G. Fan X. Liu J. Yan C. (2014). Investigation of the electrospun carbon web as the catalyst layer for vanadium redox flow battery. J. Power Sources 270, 634–645. 10.1016/j.jpowsour.2014.07.161

[B27] Xia L. Zhang Q. Wu C. Liu Y. Ding M. Ye J. (2019). Graphene coated carbon felt as a high-performance electrode for all vanadium redox flow batteries. Surf. Coat. Tech. 358, 153–158. 10.1016/j.surfcoat.2018.11.024

[B28] Xiao S. Yu L. Wu L. Liu L. Qiu X. Xi J. (2016). Broad temperature adaptability of vanadium redox flow battery—Part 1: electrolyte research. Electrochim. Acta 187, 525–534. 10.1016/j.electacta.2015.11.062

[B29] Yamamura T. Watanabe N. Yano T. Shiokawa Y. (2005). Electron-transfer kinetics of Np^3+^/Np^4+^, ${\text{NpO}}_{2}^{+}$ / ${\text{NpO}}_{2}^{2+}$, V^2+^/V^3+^, and VO^2+^/VO${}_{2}^{+}$ at carbon electrodes. J. Electrochem. Soc. 152, A830–A836. 10.1149/1.1870794

[B30] Ye J. Cheng Y. Sun L. Ding M. Wu C. Jia C. (2019). A green SPEEK/lignin composite membrane with high ion selectivity for vanadium redox flow battery. J. Membrane Sci. 572, 110–118. 10.1016/j.memsci.2018.11.009

[B31] Ye J. Wu C. Qin W. Zhong F. Ding M. (2020a). Advanced sulfonated poly (Ether Ketone)/graphene-oxide/titanium dioxide nanoparticle composited membrane with superior cyclability for vanadium redox flow battery. J. Nanosci. Nanotechnol. 20, 4714–4721. 10.1166/jnn.2020.18503 32126646

[B32] Ye J. Zhao X. Ma Y. Su J. Xiang C. Zhao K. (2020b). Hybrid membranes dispersed with superhydrophilic TiO_2_ nanotubes toward ultra-stable and high-performance vanadium redox flow batteries. Adv. Energy Mater. 10:1904041 10.1002/aenm.201904041

[B33] Yi Q. Liu Y. Zhao H. Zhou X. Liu X. Song H. (2003). Effects of acidity, temperature and surfactants on electrochemical behavior of V^5+^ ion in sulfuric acid solutions. T. Nonferr. Metal. Soc. 13, 1465–1471. Available online at: http://www.cqvip.com/qk/85276x/200306/8999397.html

[B34] Yu L. Lin F. Xiao W. Xu L. Xi J. (2019). Achieving efficient and inexpensive vanadium flow battery by combining Ce_x_Zr_1−x_O_2_ electrocatalyst and hydrocarbon membrane. Chem. Eng. J. 356, 622–631. 10.1016/j.cej.2018.09.069

[B35] Zha Q. (2002). Introduction to the Kinetics of Electrode Process, 3rd Edn. Beijing: Science and Technology Education Press.

[B36] Zhang H. (2014). Flow Battery Technology. Beijing: Chemical Industry Press.

[B37] Zhao P. Zhang H. Zhou H. Chen J. Gao S. Yi B. (2006). *In situ* surface enhanced Raman spectroscopic studies of solid electrolyte interphase formation in lithium ion battery electrodes. J. Power Sources 162, 1416–1420. 10.1016/j.jpowsour.2006.08.016

[B38] Zheng Q. Li X. Cheng Y. Ning G. Xing F. Zhang H. (2016). Development and perspective in vanadium flow battery modeling. Appl. Energy 132, 254–266. 10.1016/j.apenergy.2014.06.077

[B39] Zhong S. Skyllas-Kazacos M. (1992). Electrochemical behaviour of vanadium(V)/vanadium(IV) redox couple at graphite electrodes. J. Power Sources 39, 1–9. 10.1016/0378-7753(92)85001-Q

